# Antipyretic, anti-inflammatory and analgesic activity of *Acacia hydaspica* R. Parker and its phytochemical analysis

**DOI:** 10.1186/s12906-015-0658-8

**Published:** 2015-04-29

**Authors:** Tayyaba Afsar, Muhammad Rashid Khan, Suhail Razak, Shafi Ullah, Bushra Mirza

**Affiliations:** Department of Biochemistry, Faculty of Biological Sciences, Quaid-i-Azam University, Islamabad, Pakistan; Center for Research in Experimental and Applied Medicine, (CREAM), Army Medical College, NUST, Pakistan

**Keywords:** *A. hydaspica*, Anti-inflammatory, Analgesic, Antipyretic, Flash chromatography

## Abstract

**Background:**

Inflammation and pain underlies several pathological conditions. Synthetic drugs used for the management of these conditions carry severe toxic effects. Globally efforts are ongoing to introduce novel medicinal plants to develop effective, economic and innocuous drugs. The current study was aimed at investigating the antipyretic, anti-inflammatory and analgesic activity of methanol extract of *A. hydaspica* aerial parts (AHM) and its active fraction. Furthermore identification and isolation of polyphenolic compounds was carried out to identify the active principles.

**Methods:**

Yeast induced pyrexia, Paw edema, acetic acid-induced writhing and hot plate test were carried out *in vivo*. HPLC-DAD analysis and combination of different chromatographic techniques, involving vacuum liquid chromatography (VLC) and flash chromatography (FC) were carried out for chemical characterization. The structural heterogeneity of flavanols was characterized by ESI- MS, ^1^H NMR, ^13^C NMR and ^2^D NMR spectroscopic analyses, and also by comparison with reported literature.

**Results:**

Oral administration of *A. hydaspica* methanol extract (AHM) and *A. hydaspica* ethyl acetate fraction (AHE), showed dose and time dependent decrease in body temperature in yeast induced pyrexia, comparable to standard, Paracetamol. AHM and AHE (150 mg/kg) significantly (*p* < 0.001) inhibit pain sensation in various pain models, i.e. acetic acid induced writhing and hot plate test. Similarly AHM and AHE demonstrated an anti-inflammatory effect in carrageenan-induced paw edema in rats and 150 mg/kg dose being distinctly more effective (91.92% inhibition). When studied on prostaglandin E_2_ (PGE_2_) induced edema in rats, AHM and AHE showed maximum inhibition of edema at 150 mg/kg after 4 h. HPLC chromatogram of AHM revealed the presence of gallic acid, catechin, rutin and caffeic acid. Chromatographic separation and structure characterization of AHE, has led to the identification of three flavan-3-ol derivative including 7-*O*-galloyl catechin, +catechin and methyl gallate, which have been reported for the first time in *A. hydaspica*.

**Conclusion:**

These results revealed that the presence of bioactive compounds in *A. hydaspica* might be responsible for the pharmacological activities, confirming the indigenous utility of *A. hydaspica* against inflammatory disorders.

## Background

For centuries people of developing countries like Pakistan, India and China, rely on traditional medicinal system for the cure of various ailments as substitute health care services due to safety and cost-effectiveness of herbal medications. In different regions of Pakistan local practice of medicinal plants for curing number of diseases is very common. In Pakistan medicinal plants prescribers called Tabib/Hakim use approximately 600–1000 medicinal plants of the country based on their experience, without scientific knowledge for the treatment of wide range of disorders [1]. Certainly local practice of plants is unrestricted in developing countries, but it is obligatory to ascertain the pharmaceutically vital agents responsible for protection against fatal diseases. Currently developed world is also inclined towards complementary and alternative medicines, specifically derived from natural source. At the present, varieties of herbal plants have been extensively used as a curative agent for various infectious diseases globally. It is anticipated that about one quarter of approved modern medicines has been derived from botanicals [2].

Inflammation, pain and pyrexia underlie several pathological conditions. Synthetic drugs, i.e. NSAIDs, opioids and corticosteroids are clinically most important drugs used for the treatment of inflammatory disorders, however their long term use may induce toxic effects including; gastrointestinal ulcers, bleeding, renal disorders etc. [3,4]. Globaly efforts are ongoing to introduce novel medicinal plants to develop effective, economic and innocuous drugs [5]. Medicinal plants are believed to be an important source of useful compounds with potential therapeutic effects. The research on plants with apparent folkloric use, as agony relievers, anti-inflammatory agents, should therefore be regarded as a prolific and a rational research strategy in the search for new anti-inflammatory drugs [6].

*A. hydaspica* R. Parker belongs to family Leguminosae. This species is reported to be common in Iran, India and Pakistan, commonly used as fodder, fuel and wood [7]. It is treated as a synonym of *A. eburnea* [8]. The bark and seeds are the source of tannins. The plant is locally used as antiseptic. The traditional healers of India use various parts of the plant for the treatment of diarrhea; the leaves and the bark are useful in arresting secretion or bleeding. The pods are helpful in removing catarrhal matter and phlegm from the bronchial tubes. The gum dispels irascibility of the skin and soothes the inflamed membranes of the pharynx, alimentary canal and genito-urinary organs (http://trade.indiamart.com/details.mp?offer=6763150691). Different species of *Acacia* were evaluated for their anti-inflammatory, antipyretic and analgesic activities in various animal models. Aqueous extract of the bark of *A. karroo* provided remarkable anti-inflammatory activity against the carrageenan and histamine induced edema and analgesic activity via acetic acid induced writhing model in experimental animals [9]. Bukhari et al. evaluated the analgesic, anti-inflammatory and antiplatelet activities of the methanol extract of *A. modesta* by using acetic acid, formalin, hot plate and carrageenan induced edema in rodents [10]. Acute (xylene- induced) and chronic (cotton pellet-induced) anti-inflammatory effects of *A. nilotica* have been investigated in rat [11]. Petroleum ether, chloroform and methanol extract of *A. comigera* were evaluated against croton oil induced dermatitis in mice [12]. Ethanol extract of the seeds of *A. suma* was evaluated against the carrageenan induced hind paw edema in rat model, whereas analgesic activity was evaluated by acetic acid induced writhing and formalin induced linking tests in mice [13]. *A. nilotica* extract showed inhibitory effect on carrageenan induced paw edema and yeast induced pyrexia in rats [14].

Till date, no pharmacological study has been conducted to evaluate the antipyretic, anti-inflammatory, and analgesic activity of *A. hydaspica,* supporting traditional uses of this plant in folklore medicine. Hence the present study was undertaken to evaluate the antipyretic, anti-inflammatory and analgesic activity of methanol extract and its derived fractions using rat model. Furthermore HPLC finger printing and compounds isolation was performed to identify the active principle compounds responsible for various pharmacological activities.

## Methods

### Plant collection

The aerial parts (bark, twigs, and leaves) of *A. hydaspica* were collected from Kirpa area Islamabad, Pakistan in the month of April 2011. After identification with the help of relevant literature, voucher specimen (0642531) was assigned and the herbarium specimen was submitted in the Herbarium of Pakistan, Museum of Natural History, Islamabad for future reference.

### Extraction

Shade dried aerial parts (bark, twigs, and leaves) of *A. hydaspica* were ground in to powder (3 kg) and soaked for 14 days in crude methanol, with occasional shaking. The extract was filtered through filter paper (Whatman filter paper number 45), and concentrated under vacuum using a rotary evaporator (Buchi, R114, Switzerland) at 40°C and 472 g of *A. hydaspica* crude methanol extract (AHM, 15.73%) was obtained. Partial purification or separation of AHM was done by solvent-solvent extraction. Briefly 12 g of AHM was suspended in 500 ml distilled water in separating funnel (1000 ml) and successively partitioned with *n-*Hexane, Ethyl-acetate, Chloroform and *n-*Butanol. Each extraction process was repeated three times with 500 ml of each solvent, same process was repeated to get enough mass of fractions for various bioactivity testing and chromatographic separation. These solvents with varying polarities theoretically partitioned different plant constituents. The filtrate was concentrated using rotary evaporator and weighed to determine the resultant mass. After this initial partitioning we got four soluble fractions; *A. hydaspica n*-hexane fraction (AHH, 5.27% yield), *A. hydaspica* ethyl acetate fraction (AHE, 27.77% yield), *A. hydaspica* chloroform fraction (AHC, 1.94% yield), *A. hydaspica n*-butanol fraction (AHB, 41.66% yield) and remaining aqueous fraction (AHA, 8.05% yield). The AHE showed to be the most active fraction and AHH, AHC, AHB and AHA fractions were not found active in any of the assays carried out in this study. AHM was subjected to High Performance Liquid Chromatography (HPLC) for compound fingerprinting and AHE was subjected to chromatographic isolation for further fractionation and purification of polyphenolics.

### Compositional analysis by HPLC

HPLC analysis of AHM was carried out by using HPLC-DAD (Agilent Germany) equipment using Sorbex RX-C8 (Agilent USA) analytical column with 5 μm particle size. Mobile phase consisted of eluent A, (acetonitrile–methanol–water–acetic acid /5: 10: 85: 1) and eluent B (acetonitrile-methanol-acetic acid/40: 60: 1). The gradient (A: B) utilized was the following: 0–20 min (0 to 50% B), 20–25 min (50 to 100% B), and then isocratic 100% B (25-40 min) at flow rate of 1 ml/min, and injection volume was 20 μl. Rutin and Gallic acid were analyzed at 257 nm, catechin and apigenin at 279 nm, caffeic acid at 325 nm and quercitin, myricetin, kaempherol were analyzed at 368 nm. Every time column was reconditioned for 10 min before the next analysis. All samples were assayed in triplicate. Quantification was carried out by the integration of the peak using the external standard method. All chromatographic operations were carried out at ambient temperature.

### Isolation of compounds

**Figure 1:**
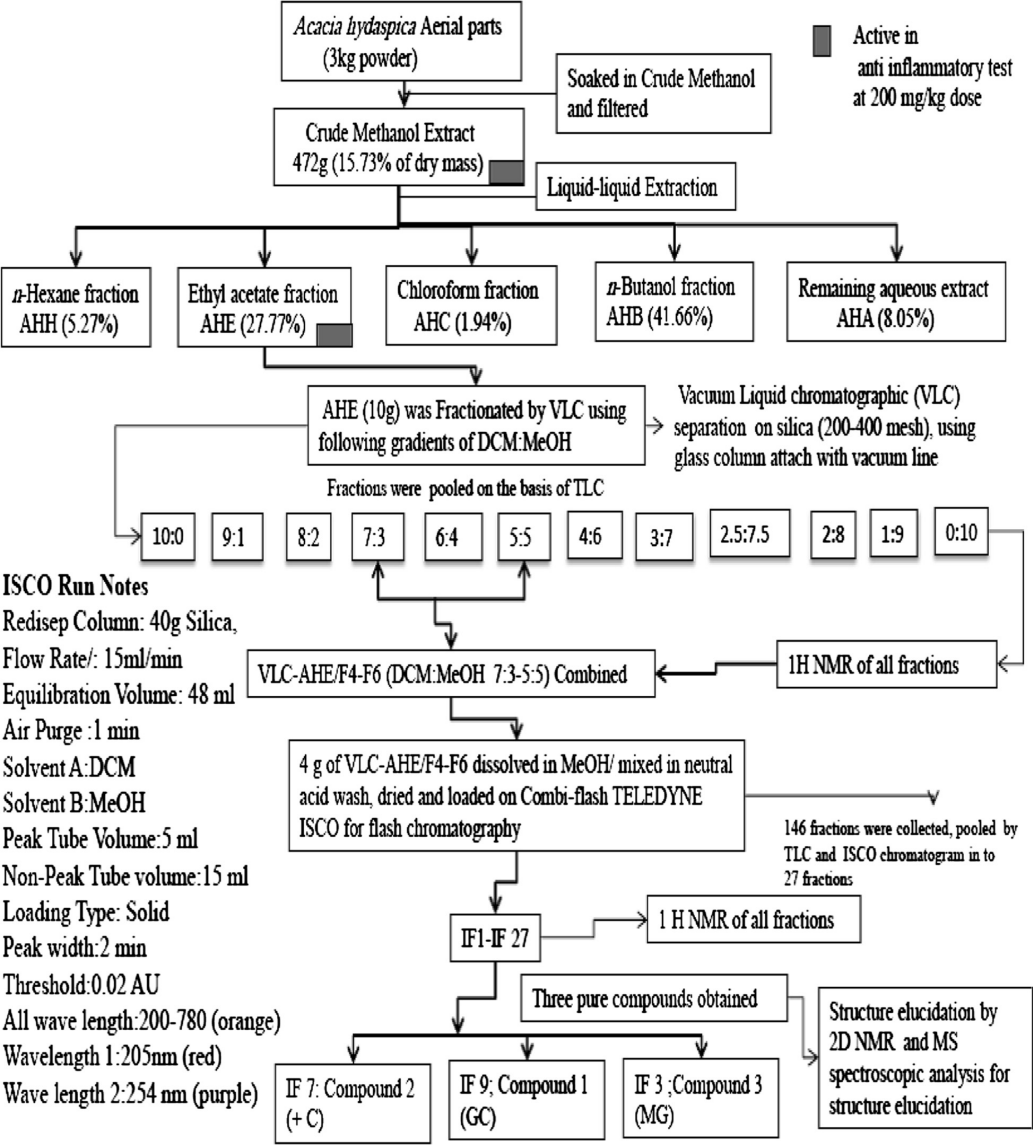
Schematic representation of extraction and isolation of compounds from *A. hydaspica*.

The scheme of fractionation and isolation is explained in flow chart (Figure 1). Briefly 10 g of *A. hydaspica* ethyl acetate fraction (AHE) was dissolved in DCM (Dichloromethane), mixed with neutral acid wash (super cell NF) and dried down completely with rotavap. Dried extract sample was loaded on silica gel using glass column packed with silica (200–400 mesh), attached with vacuum line source (vacuum liquid chromatography, VLC). The column was eluted with dichloromethane (DCM), then DCM-methanol mixture in increasing order of polarity and 10 fractions were collected (1 L each), and three major phenolic fractions (VLC-AHE/F4-F6) selected on the basis of TLC (silica gel 60 F_254_ plates, MERCK) and ^1^HNMR spectra similarity, and were combined, and subjected to flash liquid chromatography, carried on Combi-flash Teledyne ISCO using silica and column eluted with mixture of DCM: Methanol in increasing order of polarity. Spectra were monitored at all wavelengths (200 nm-780 nm) with Peak width 2 min, and Thresh hold 0.02 AU. 146 fractions collected with ISCO were pooled into 27 fractions according to their TLC and ISCO chromatogram spectral peaks. ^1^HNMR of ISCO fractions indicated the presence of chromatographically pure compounds IF 9 (C1), IF7 (C2) and IF3 (C3); Structures of isolated compounds were determined by NMR assignments.

### Nuclear magnetic resonance spectroscopy (NMR)

^1^H- and ^13^C-NMR spectrum for all compounds was recorded on a CDD NMR instrument: Varian 600 MHz (^1^H and ^13^C frequencies of 599.664 and 150.785 MHz, respectively) at 25°C. Spectra of all compounds were obtained in Methanol-d4 and DMSO-d6. Conventional 1D and 2D Fourier transform techniques were employed as necessary to achieve unequivocal signal assignments and structure proof for all compounds independently. Stereo-chemical assignments were made with ROESY and NOESY experiments. Detailed analysis of resolution enhanced spectra (Peak picking, integration and multiplet analysis) was performed using Varian NMR and ACD/NMR processor, Academic Edition. The NMR spectra and chemical shifts of isolated compounds were compared with published data.

### Animals

Sprague Dawley rats (180-220 g) of either sex, maintained at primate facility Quaid-i-Azam University Islamabad, Pakistan were used for the study. Animals were kept at standard conditions of temperature (25 ± 1°C) and 12/12 h light/dark cycle, have free access to standard laboratory feed and water. All experimental procedures involving animals were conducted in accordance with the guidelines of the National Institutes of Health (NIH guidelines). The study protocols were approved by Ethical committee of Quaid-i-Azam University Islamabad. Six animals were used in each group. Experiments on animals were performed in accordance with the guidelines of the institute of animal ethical committee, NIH, Islamabad.

### Acute toxicity study

The acute toxicity studies were conducted as per the guidelines 425 of Organization for Economic Cooperation and Development (OECD) for testing of chemicals for acute oral toxicity [15]. Rats (n = 6) of either sex treated with different doses (50, 250, 500, 1000, 2000 and 3000 mg/kg, p.o.), while the control group received saline (10 ml/kg). All the groups were observed up to 6 h for any gross effect and then mortality rate was observed after 24 h of treatment.

### Pharmacological activities

#### Antipyretic activity

The antipyretic activity of AHM and its active fraction AHE was evaluated using Sprague Dawley rats (180–220 g) of either sex using previously explained method [16]. The selected animals were healthy and normal body temperature of each rat was checked by using digital thermometer. Pyrexia was induced in all rats by injection of 20% aqueous suspension of brewer’s yeast (*Saccharomyces cerevisiae*) 10 ml/kg. Subcutaneous (SC). All animal groups (6/group) were fasted with access to only water, after injection of yeast for 24 h. After that, the rectal temperature of each rat was recorded and pyrexia was confirmed by increase in temperature more than 1°C, while animals showing less than 1°C rise in temperature were excluded from the experiment. The group I received saline (10 ml/kg), group II received paracetamol (100 mg/kg) as a standard drug, while group III-XII received 50, 100 and 150 mg/kg, p.o. doses (through feeding tubes) of AHM and AHE respectively. The rectal temperature of the groups was recorded at 1 h intervals for 5 h.

#### Anti-inflammatory activity

##### Carrageenan induced paw edema

The anti-inflammatory activity was performed on rat of either sex (180-220 g). The normal paw volumes of all the rats were measured ab initio and animals were divided into different groups, each consisting of six rats [1]. The group I was treated with normal saline (10 ml/kg, i.p.), Group II, III and IV with the standard drugs i.e. diclofenac sodium, aspirin and fluoxetine (10 mg/kg) respectively while rest of the groups were treated with AHM and AHE (50, 100 and 150 mg/kg, p.o.). After thirty minutes of the above intra-peritoneal and oral administration, carrageenan (1%, 0.1 ml) was given subcutaneously into the sub plantar tissue of the right hind paw of each rat. The paw volume was quantified using digital plethysmometer before and at 1^st^, 2^nd^, 3^rd^ and 4^th^ h after carrageenan administration. The edema volume of paw and percent inhibition of edema were calculated using the following formulae:

$$ \mathrm{E}\mathrm{V}=\mathrm{P}\mathrm{V}\mathrm{A}-\mathrm{P}\mathrm{V}\mathrm{I} $$

EV = edema volume, PVI = Paw volume before carrageenan administration (i.e. initial paw volume) and, PVA = Paw volume after carrageenan administration.

$$ \mathrm{Percent}\kern0.5em \mathrm{inhibition}=\left[\frac{\mathrm{EVc}-\mathrm{E}\mathrm{V}\mathrm{t}\Big)}{\mathrm{EVc}} \times 100\right] $$

EVc = Edema volume of control animals, EVt = Edema volume of test drug animals.

##### Prostaglandin E_2_-induced paw edema

Rats of either sex were divided into different groups (n = 6) and treated intraperitoneal with saline (control), Diclofenac Sodium (10 mg/kg, i.p., reference standard), AHM and AHE (50, 100 and 150 mg/kg orally). After 30 min of treatment, 100 μl of prostaglandin E_2_ (0.01 μl/ml) was administered into the sub planter side of right hind paw of each rat, and the edema size was determined as aforesaid [17].

#### Analgesic activity

##### Acetic acid induced writhing test

The method used in this test has previously been described by khan et al. [18]. Total number of writhing movements following i.p. administration of acetic acid solution (10 ml/kg, 1%) was recorded over a period of 10 min, starting 5 min after acetic acid injection. Rats were treated with AHM and AHE (50, 100 and 150 mg/kg), vehicle (saline), standard drugs (diclofenac sodium and aspirin 10 mg/kg), 30 min before acetic acid injection [19]. The numbers of writhings movements (Constriction of abdominal muscles along with the stretching of hind limbs) were counted in both untreated and treatment groups and percentage inhibition in abdominal writhings was calculated as follow.

$$ \%\kern0.5em \mathrm{inhibition}\kern0.5em \mathrm{of}\kern0.5em \mathrm{abdominal}\kern0.5em \mathrm{writhing}=\left[\frac{\mathrm{Wc}-\mathrm{W}\mathrm{t}\Big)}{\mathrm{Wc}} \times 100\right], $$

W = No. of writhing, c = Control, and t = Test.

##### Hot plate test

The procedure described by Muhammad et al. [1], was followed to perform this test. Sprague Dawley rats of either sex (n = 6) weighing 180–220 g were used. Animals were subjected to pre-testing on a hot plate (Harvard apparatus) maintained at 55 ± 0.1°C. Animals having latency time greater than 15 sec on hot plate during pre-testing were excluded. Animals were divided randomly into 5 groups, each containing of six rats. The group I was treated with saline (10 ml/kg), group II and III with diclofenac sodium and fluoxetine (10 mg/kg, i.p.), and Group IV-IX were treated with oral doses of 50, 100 and 150 mg/kg of AHM and AHE respectively. Diclofenac sodium and fluoxetine were used as reference drugs for comparison [19,20]. After 30 min of dose administration, rats were dropped inside the cylinder onto the hot plate and the latency time (time for which rat remains on the hot plate without licking or flicking of hind limb or jumping) was recorded in seconds. In order to prevent the tissue damage the cut off time of 30 sec was set for all animals. The latency time was recorded for each group at 0, 30, 60, 90 and 120 min following drug administration. Percent analgesia was calculated using the following formula.

$$ \%\kern0.5em \mathrm{Analgesia}=\left[\frac{\left(\mathrm{Test}\ \mathrm{latency}\ \hbox{--}\ \mathrm{control}\ \mathrm{latency}\right)\ }{\left(\mathrm{Cut}\ \mathrm{off}\ \mathrm{time}-\mathrm{control}\ \mathrm{latency}\right)} \times 100\right] $$

#### Statistical analysis

All values were expressed as mean ± SEM and data were analyzed by Graph pad using One-way analysis of variance followed by Tukey’s multiple comparison test and Two-way analysis of variance (ANOVA) followed by Bonferroni multiple comparison test. *p* < 0.05 was considered significant.

## Results

### Estimation of acute toxicity

AHM and AHE found safe at all tested doses (up to 3000 mg/kg) and did not show any noxious symptom in rats like sedation, convulsions, diarrhea, and irritation. During the 48 h assessment, no mortality was found.

### Antipyretic activity

The AHM and AHE significantly (*p* < 0.001) attenuated hyperthermia in rats. The mean increase in rectal temperature recorded after 24 h of yeast injection, was 2°C-2.43°C. The inhibition was dose dependent and remained significant up to 5 h of administration. AHE 150 mg/kg dose showed the maximum antipyretic effect and return body temperature to normal levels (*p* > 0.05) more efficiently than standard drug paracetamol (Table 1).

**Table 1:** **Effect of AHM and its fraction on brewer’s yeast-induced pyrexia**

**Treatment**	**Dose mg/kg**	**Body temp. (N)**	**Rectal temperature (°C)**					
			**After administration of drug**					
			**After 24 h (T)**	**1 h (P1)**	**2 h (P2)**	**3 h (P3)**	**4 h (P4)**	**5 h (P5)**
**Saline**	10 ml	36.96 ± 0.21	38.96 ± 0.02	38.88 ± 0.09	38.73 ± 0.03	38.62 ± 0.02	38.55 ± 0.06	38.5 ± 0.014
**Paracetamol**	100	37.36 ± 0.25	39.35 ± 0.21	38.70 ± 0.27	37.99 ± 0.07*	37.79 ± 0.04*	37.49 ± 0.02**	37.395 ± 0.02***
**AHM**	50	37.23 ± 0.12	39.03 ± 0.06	39.32 ± 0.12	38.47 ± 0.084	38.15 ± 0.05	37.91 ± 0.03	37.77 ± 0.14
	100	36.85 ± 0.05	39.17 ± 0.08	39.05 ± 0.08	38.12 ± 0.021	37.94 ± 0.06	37.76 ± 0.05*	37.12 ± 0.03***
	150	37.30 ± 0.1	39.53 ± 0.18	39.20 ± 0.14	38.05 ± 0.09***	37.86 ± 0.04***	37.62 ± 0.03*	37.36 ± 0.06***
**AHE**	50	37.19 ± 0.10	39.43 ± 0.54	38.82 ± 0.41	38.01 ± 0.014	37.99 ± 0.01	37.68 ± 0.045*	37.24 ± 0.239***
	100	36.85 ± 0.05	38.67 ± 0.44	38.6 ± 0.489	37.88 ± 0.03*	37.475 ± 0.324**	37.31 ± 0.19***	36.75 ± 0.245***
	150	37.10 ± 0.10	39.18 ± 0.28	38.55 ± 0.45	37.62 ± 0.02**	37.625 ± 0.494**	37.195 ± 0.005***	37.13 ± 0.12***

Data values shown represent mean ± SEM (n = 6). *p < 0.05, **p < 0.01, ***p < 0.001 versus only brewer’s yeast treated group (Two-way ANOVA followed by Bonferroni multiple comparison test). N indicates mean body temperature before pyrexia.

### Anti-inflammatory activity

#### Carrageenan induced paw edema

In the carrageenan-induced edema, crude extract of *A. hydaspica* (AHM) and its fraction (AHE), induced a dose and time dependent reduction in paw edema. The anti-inflammatory activity became significant (*p* < 0.05), 2 h after carrageenan injection by 150 mg/kg dose whilst; maximum inhibition was observed after 4 h. The percent inhibition of inflammation by AHM (150 mg/kg) was more pronounced than aspirin, whilst the inflammatory activity revealed by diclofenac sodium was non-significantly different from AHM (Table 2).

**Table 2:** **Effect of AHM and its fraction on carrageenan-induced paw edema in rats**

**Treatment**	**Dose/route**	**Mean paw volume before carrageenan injection**	**Increase in paw volume (ml) after carrageenan injection (mean ± SEM)/Percent inhibition of edema**			
			**+1 h**	**+2 h**	**+3 h**	**+4 h**
**Saline**	2 ml, i.p.	1.2 ± 0.0265	2.66 ± 0.015	2.5 ± 0.095	2.24 ± 0.104	1.93 ± 0.053
**AHM**	50 mg/kg, p.o.	1.043 ± 0.03	2.488 ± 0.045	1.82 ± 0.062	1.56 ± 0.047	1.39 ± 0.032
			(1.34 ± 0.917)	(40.23 ± 0.413)	(52.29 ± 3.12)	(52.46 ± 2.419)
	100 mg/kg, p.o.	0.89 ± 0.04	2.311 ± 0.03	1.543 ± 0.02	1.301 ± 0.015	1.156 ± 0.05
			(3.11 ± 1.6)	(49.41 ± 2.023)	(60.47 ± 0.429*)	(63.47 ± 0.457*)
	150 mg/kg, p.o.	1.01 ± 0.037	2.423 ± 0.043	1.49 ± 0.07	1.27 ± 0.060	1.119 ± 0.06
			(3.63 ± 1.135)	(63.07 ± 0.09*)	(75 ± 0.23**)	(85.06 ± 1.47**)
**AHE**	50 mg/kg, p.o.	1.05 ± 0.05	2.473 ± 0.013	1.797 ± 0.039	1.473 ± 0.046	1.343 ± 0.029
			(2.295 ± 0.10)	(46.64 ± 0.63***)	(69.56 ± 0.564***)	(78.29 ± 1.000***)
	100 mg/kg, p.o.	1.01 ± 0.29	2.307 ± 0.036	1.491 ± 0.010	1.274 ± 0.37	1.09 ± 0.51
			(2.50 ± 0.20)	(67.14 ± 0.500***)	(81.159 ± 0.5***)	(92.07 ± 1.00***)
	150 mg/kg, p.o.	1.01 ± 0.5	2.403 ± 0.05	1.46 ± 0.026	1.178 ± 0.019	1.036 ± 0.036
			(3.86 ± 0.428)	(67.85 ± 1.00***)	(87.82 ± 1.00***)	(93.40 ± 1.00***)
**Diclofenac sodium**	10 mg/kg, i.p.	1.19 ± 0.015	2.576 ± 0.03	1.600 ± 0.02	1.426 ± 0.0305	1.27 ± 0.01
			(5.52 ± 0.575)	(68.46 ± 0.815**)	(77.24 ± 0.192**)	(89.04 ± 2.048***)
**Fluoxetine**	10 mg/kg, i.p.	1.09 ± 0.064	2.50 ± 0.091	1.63 ± 0.04	1.343 ± 0.055	1.192 ± 0.017
			(3.86 ± 0.212)	(58.461 ± 1.972*)	(75.64 ± 0.093**)	(85.98 ± 2.008**)
**Aspirin**	10 mg/kg, i.p.	1.157 ± 0.04	2.597 ± 0.032	1.736 ± 0.075	1.486 ± 0.066	1.34 ± 0.0264
			(1.84 ± 0.50)	(55.46 ± 0.246*)	(68.30 ± 0.094**)	(74.93 ± 5.15**)

Data values shown represent mean ± SEM (n = 6). *p < 0.05, **p < 0.01, ***p < 0.001 versus only carrageenan treated group (Two-way ANOVA followed by Bonferroni multiple comparison test). Percentage inhibition is shown in brackets. Inhibition in saline treated group at each time point was calculated relative to paw edema after 1 h.

#### Prostaglandin induced paw edema

AHM and AHE revealed a dose dependent (50-15 mg/kg) and time dependent reduction in prostaglandin E_2_ (PGE_2_) induced paw edema in rats. Reversal of edema starts 2 h after the injection of PGE_2_, and peak inhibition was seen after 4 h. The AHE induced anti-inflammatory activity comparable to standard reference drug (Table 3).

**Table 3:** **Effect of AHM and its fraction on prostaglandin E**_**2**_ **-induced paw edema in rats**

**Treatment**	**Dose/route**	**Percent inhibition of edema Volume**			
		**+1 h**	**+2 h**	**+3 h**	**+4 h**
**AHM**	50 mg/kg, p.o.	2.44 ± 0.29	18.58 ± 1.744***	52.25 ± 1.52***	54.186 ± 3.13***
	100 mg/kg, p.o.	3.76 ± 0.176	33.31 ± 1.084***	72.86 ± 2.081***	75.19 ± 2.62***
	150 mg/kg, p.o.	4.16 ± 0.176	42.50 ± 1.76***	86.72 ± 3.45***	86.11 ± 1.41***
**AHE**	50 mg/kg, p.o.	4.72 ± 0.355	24.16 ± 1.20***	64.34 ± 1.88***	75.68 ± 2.61***
	100 mg/kg, p.o.	4.33 ± 0.12	37.77 ± 1.36***	77.21 ± 2.23***	84.12 ± 0.90***
	150 mg/kg, p.o.	4.80 ± 0.152	59.338 ± 3.05***	90.57 ± 0.76***	91.345 ± 0.57***
**Diclofenac sodium**	10 mg/kg, i.p.	5.13 ± 0.145	55.83 ± 2.63***	87.72 ± 2.53***	88.11 ± 0.68***

Data values shown represent mean ± SEM (n = 6). ***p < 0.001 versus only PGE_2_ treated group (Two-way ANOVA followed by Bonferroni multiple comparison test).

### Peripheral analgesic effect

#### Acetic acid induced writhing test

Results showed that AHM and its derived fraction AHE significantly (*p* < 0.001) and dose dependently (50, 100 and 150 mg/kg), reduced the number of abdominal constriction induced by administration of 1% acetic acid solution. The inhibitory effect of Diclofenac sodium was non-significantly different from the protective effect revealed by AHM and AHE (100-150 mg/kg dose) (Table 4).

**Table 4:** **Effect of AHM and its fraction on acetic acid induced writhing**

**Groups**	**Drug (dose), route**	**No. of writhing (mean ± SEM)**	**% inhibition**
**Saline**	10 ml/kg	70.38 ± 2.7	0
**AHM**	50 mg/kg,p.o.	27.65 ± 1.005***	60.71 ± 0.66
	100 mg/kg, p.o.	18.09 ± 1.24***	74.29 ± 1.20
	150 mg/kg, p.o.	16.15 ± 1.175***	77.05 ± 1.70
**AHE**	50 mg/kg,p.o.	18.25 ± 1.25***	74.06 ± 1.15
	100 mg/kg, p.o.	15.00 ± 1.00***	78.68 ± 1.05
	150 mg/kg, p.o.	12.75 ± 1.25***	81.88 ± 0.05
**Diclofenac sodium**	10 mg/kg, i.p.	13.22 ± 1.89***	81.65 ± 1.10
**Aspirin**	10 mg/kg, i.p.	20.13 ± 1.12***	71.39 ± 2.00

Data values shown represent mean ± SEM (n = 6). ***p < 0.001 versus only acetic acid treated group (One-way ANOVA followed by Tukey’s Multiple Comparison Test).

### Central analgesic effect

#### Hot plate method (Thermal stimulation)

**Figure 2:**
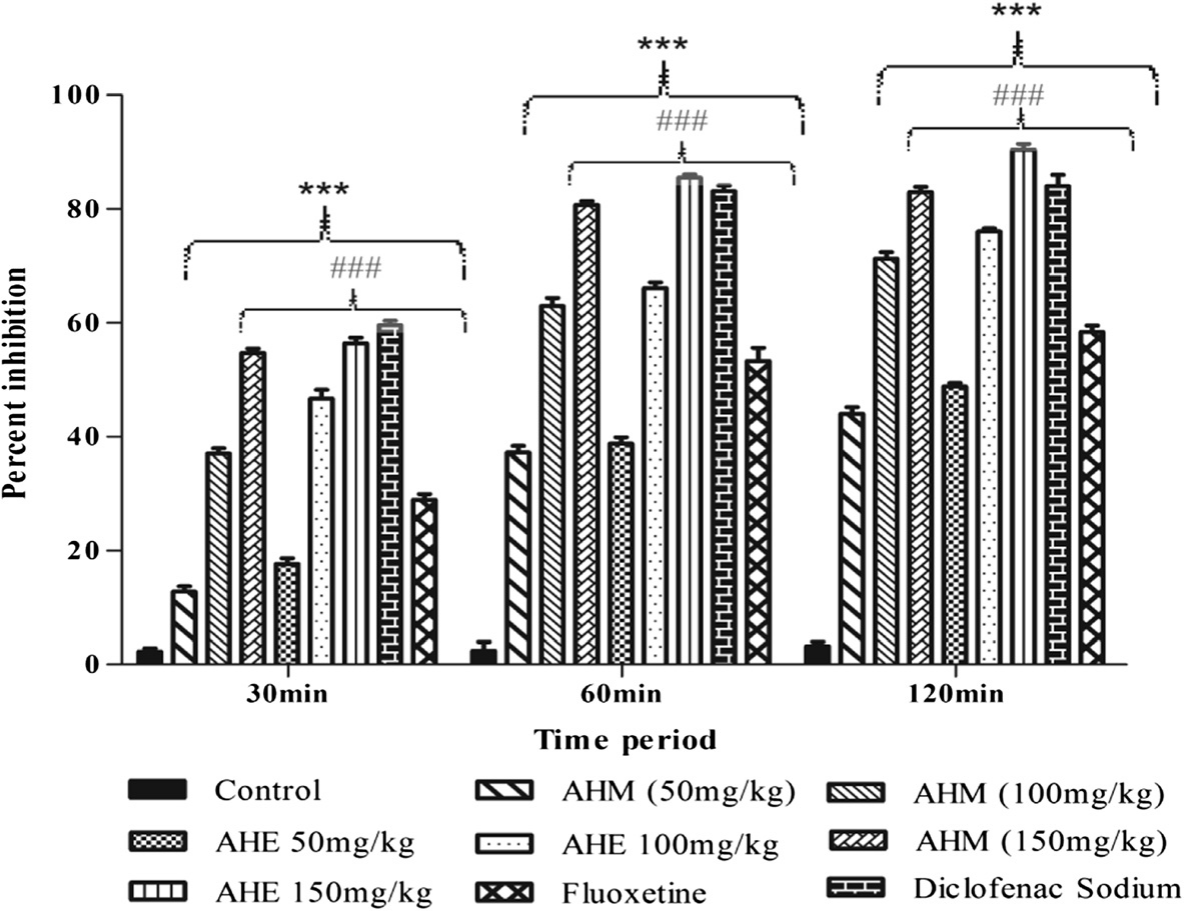
Percent effect of AHM, AHE, diclofenac sodium and fluoxetine in hot plate test. Data analyzed by Two-way ANOVA followed by Bonferroni comparison test. Asterisks *** indicated statistically significant (*p* < 0.001) values from the control, ### indicated statistically significant (*p* < 0.001) difference of AHM and AHE (150 mg/kg dose) to fluoxetine.

In this assay *A. hydaspica* methanol extract (AHM) and its ethyl acetate fraction (AHE) exhibited a dose dependent increase in latency time and inhibited pain sensation in a pattern similar to standard drug; diclofenac sodium whilst the effect of both AHM and AHE, was shown to be more pronounced than fluoxetine at 150 mg/kg dose (Figure 2, Table 5).

**Table 5:** **Effect of AHM and its fraction in hot plate test**

**Group**	**Dose/route**	**Latency time in seconds**			
		**0 min**	**30 min**	**60 min**	**120 min**
**Saline**	10 ml, i.p.	9.83 ± 0.601	9.36 ± 0.317	9.3. ± 0.881	9.167 ± 0.44
**AHM**	50 mg/kg, p.o.	9.61 ± 0.1675	12.33 ± 0.881**	17.00 ± 0.577***	18.33 ± 0.66***
	100 mg/kg, p.o.	9.76 ± 0.145	17.0 ± 0.57***	22.334 ± 1.201***	24.0 ± 0.557***
	150 mg/kg, p.o.	9.74 ± 0.52	20.66 ± 0.881***	26.0 ± 0.577***	26.33 ± 0.334***
**AHE**	50 mg/kg, p.o.	9.63 ± 0.185	13.0 ± 0.577***	17.33 ± 0.667***	19.33 ± 0.333***
	100 mg/kg, p.o.	9.83 ± 0.167	17.66 ± 0.88***	23.0 ± 0.577***	24.33 ± 0.333***
	150 mg/kg, p.o.	9.667 ± 0.166	22.0 ± 0.577***	26.66 ± 0.33***	27.0 ± 0.577***
**Diclofenac sodium**	10 mg/kg, i.p.	9.67 ± 0.667	21.66 ± 0.88***	26.16 ± 0.89***	26.66 ± 1.00***
**Fluoxetine**	10 mg/kg, i.p.	9.66 ± 0.881	15.33 ± 0.334***	20.33 ± 0.90***	21.33 ± 0.667***

Data values shown represent mean ± SEM (n = 6). **p < 0.01, ***p < 0.001 versus saline treated group (Two-way ANOVA followed by Bonferroni multiple comparison test).

### HPLC analysis

**Figure 3:**
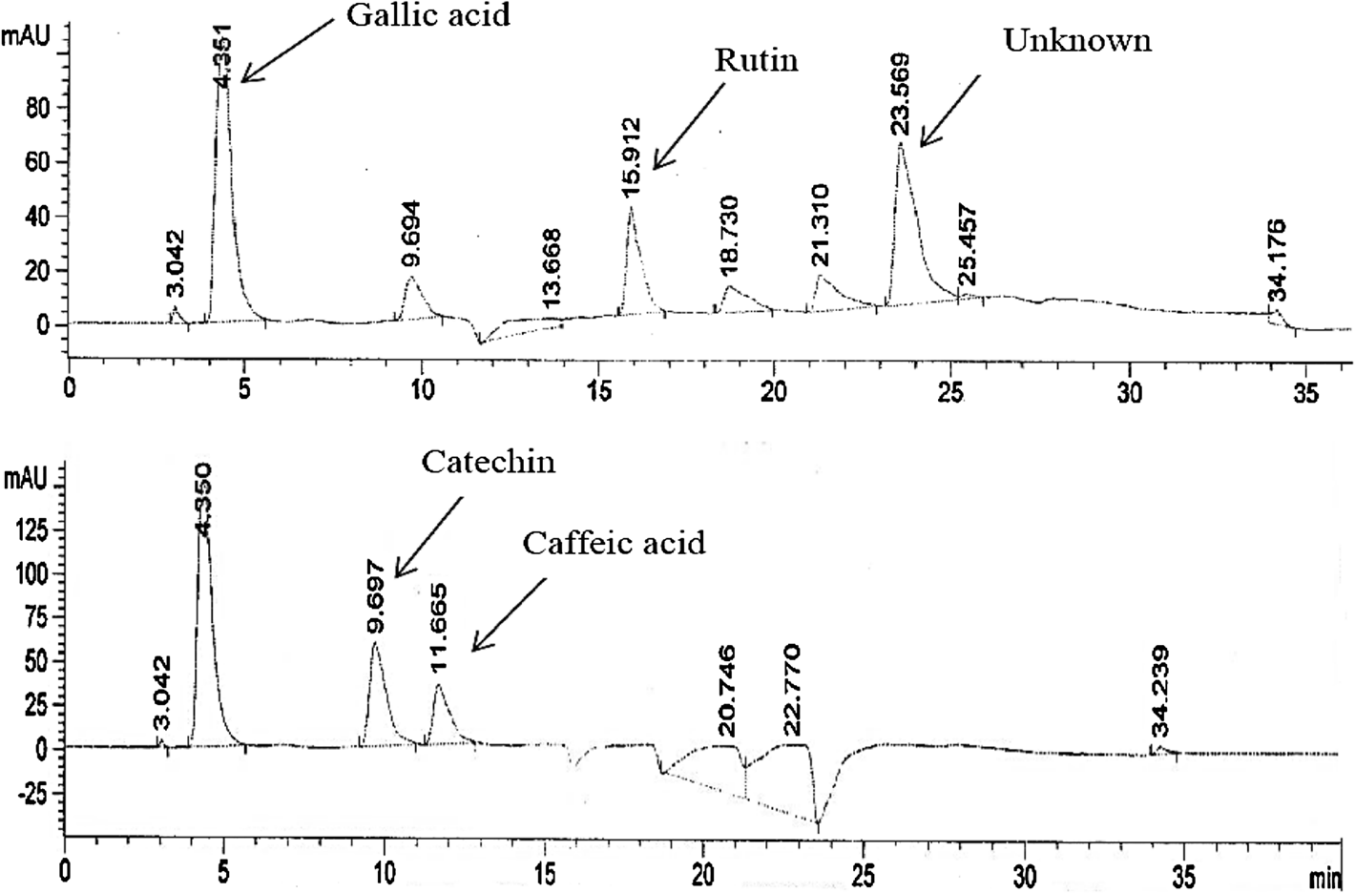
HPLC chromatogram of AHM reveals the presence of Gallic acid, rutin, catechin and Caffeic acid.

To establish the fingerprint chromatogram for AHM, Gallic acid, catechins, caffeic acid, rutin, apigenin, kaempferol, myricetin and quercetin were used as markers. HPLC chromatogram revealed the presence of gallic acid, catechin, caffeic acid, and rutin by comparison with retention time and UV absorbance of purified standards. The relative amounts of the four phenolic compounds found in AHM were in the order of catechin (558.9 μg/100 mg dry sample) *>* Gallic acid (300.051 μg/100 mg dry sample) *>* rutin (235.38 μg /100 mg dry sample) *>* caffeic acid (137.43 μg/100 mg dry sample), respectively (Figure 3).

### Phytochemistry of AHE

**Figure 4:**
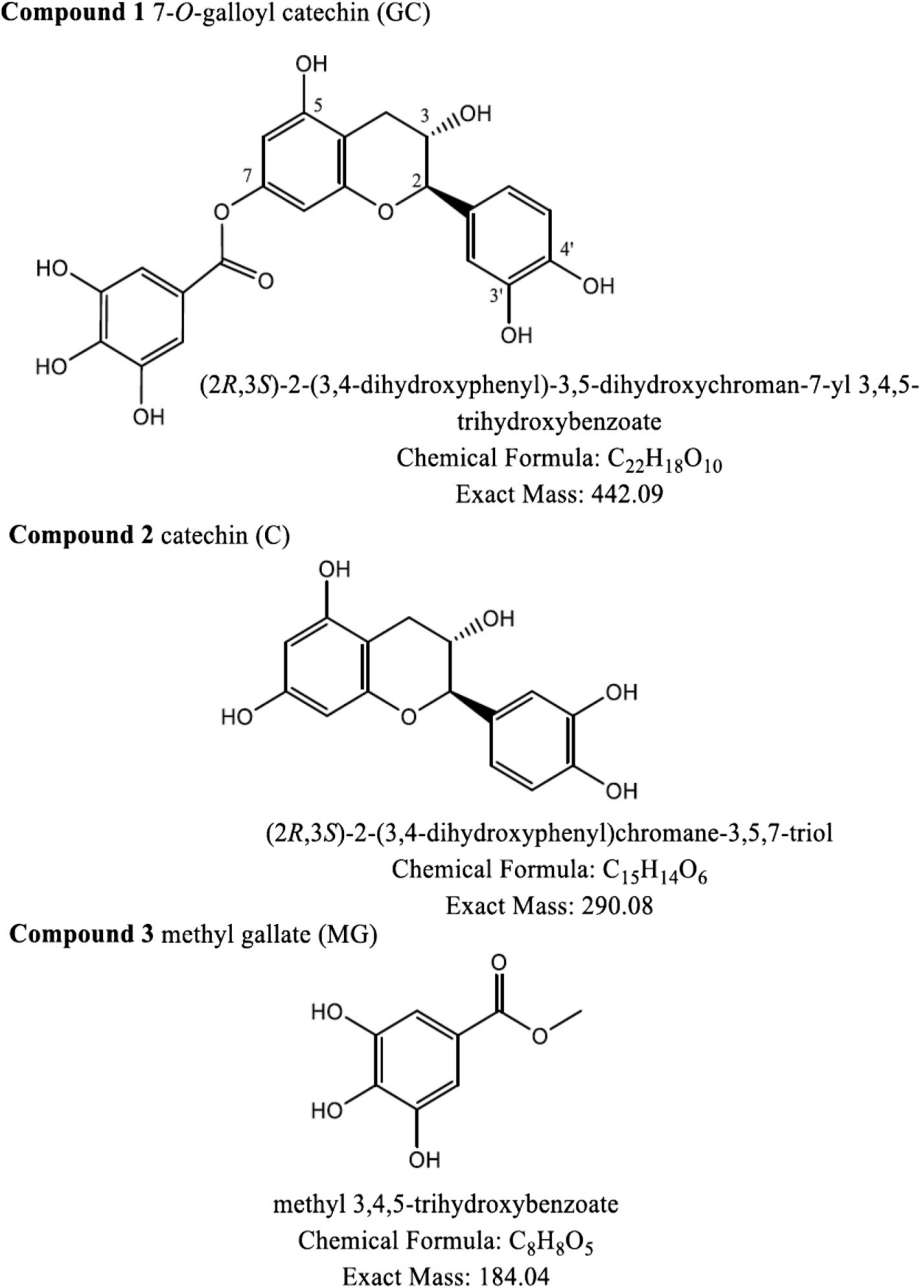
Chemical structures of isolated polyphenols from *A. hydaspica* ethyl acetate fraction (AHE).

The ethyl-acetate fraction of *A. hydaspica* methanol extract was fractionated by VLC chromatography and ISCO flash chromatography to afford several enriched fractions and three pure compounds C1, C2 and C3. Isolated compounds were identified as 7-*O*-galloyl catechin (**C1**), catechin (C**2**) [20,21] and methyl gallate (C**3**) [22] by comparison of their 1D and 2D NMR spectral data with the reported data in the literature. Figure 4 has shown the structure of isolated flavanols from AHE. *A. hydaspica* ethyl-acetate extract (AHE) yields 187.5 mg/g of Compound 1, 100 mg/g of Compound 2 and 37.5 mg/g of Compound 3 (Figure 4).

## Discussion

To the best of our knowledge this is the first report on antipyretic, analgesic and anti- inflammatory activities of *A. hydaspica* methanol extract of arial parts and its derived ethyl acetate fraction (AHE). Present investigation showed that the AHM and AHE possess noticeable antipyretic, analgesic and anti-inflammatory properties with a reasonable protection profile.

HPLC analysis of AHM extract shows the presence of bioflavonoids, Gallic acid, catechin, caffeic acid and rutin, which were identified for the first time in subject plant. Furthermore chemical investigation of AHE resulted in isolation of 7-*O*-galloyl catechin, +catechin and methyl gallate.

Gallic acid revealed anti-inflammatory activity by interfering with the functioning of polymorphonuclear leukocytes (PMNs) and assembly of active NADPH-oxidase. Presence of Gallic acid in AHM might be contributing to strong anti-inflammatory activity as, O-dihydroxy group of gallic acid is important for the inhibitory activity in vitro [23]. Caffeic acid exerted both *in vitro* and *in vivo* anti-inflammatory effects probably through modulation of iNOS expression and other inflammatory mediators. Catechin is the class of flavonoids with potent cancer chemo-preventive, neuro-protective, anti-apoptotic, and anti-inflammatory properties in clinical disorders [24,25]. Rutin is an important plant secondary metabolite and reported as hepato-protective, antioxidant, and anti-inflammatory agent [25].

AHE was also found to possess the most significant pharmacological activities, which might be due to the presence of isolated flavanols: 7-*O*-galloyl catechin, +catechin and methyl gallate, previous data also affirmed that these compounds possess anti inflammatory, analgesic and pain relieving potential [26].

A subcutaneous injection of Brewer’s yeast induces pyrexia (called as pathogenic fever) by increasing the production of prostaglandins. Antipyretic activity is commonly mentioned as a characteristic of drugs or compounds which have an inhibitory effect on prostaglandin-biosynthesis and it is considered as a useful test for the screening of plant materials as well as synthetic drugs for their antipyretic potential [1]. AHM and its fraction AHE exhibited significant antipyretic activity at 150 mg/kg dose, comparable to paracetamol (standard drug). Inhibition of prostaglandin synthesis by blocking the cyclooxygenase enzyme activity could be the possible mechanism of antipyretic action as that of paracetamol. Besides that antipyretic effect might be governed by the ability of extract samples to reduce pro-inflammatory mediators, improve anti-inflammatory signals at sites of injury, or increase antipyretic messages within the brain [27]. The observed effects might be due to the presence of pharmacologically active metabolites that might interfere with the release of prostaglandins. However, it must be noted that several biochemical events occur ultimately to the production of prostaglandins. It may therefore be worthwhile to investigate the exact point in the biochemical events where the extract exerts its antipyretic effect.

Carrageenan-induced paw edema is an acceptable preliminary screening test for anti-inflammatory activity [28]. Carrageenan induces paw edema bi-phasically: the initial phase extending from 0–2.5 h, predominantly results due to the release of histamine, serotonin and bradykinin. However, COX enzyme is known to play a key role in the development of the later phase of inflammation by converting arachidonic acid into prostaglandins. This enzyme is considered to be identified target for a variety of NSAIDs, such as aspirin and diclofenac sodium, which inhibit rat paw edema at the later phase following carrageenan injection [29-31]. Therefore for comparison three standards were used. AHM significantly (*p* < 0.001) inhibited paw edema in the later phase in a pattern similar to diclofenac sodium, whose mechanism of action is inhibition of cyclooxygenase enzyme synthesis. Although the actual mechanism of action is not known, it is possible that, the anti-inflammatory activity exhibited by *A. hydaspica* extracts could be attributed to the inhibition of the synthesis, release or action of inflammatory mediators. Therefore; we tested AHM and AHE extracts against PGE_2_ induced paw edema in rats. Results showed that both extracts significantly reduced PGE_2_ induced edema with maximum protection observed after 4 h. PGE_2_ is an important mediator of second phase inflammation. These results are in concordance with reported literature for other *Acacia* species which possesses anti-asthmatic, analgesic, anti-inflammatory, and antioxidant properties [32]. The inhibition of inflammation by extracts could be attributed to the presence of active constituents.

Acetic acid induced writhing test is well proposed method in evaluating the medicinal agents for the analgesic potential. Pain sensation in acetic acid induced writhing paradigm is elicited by producing a localized inflammatory response due to the release of free arachidonic acid from tissue phospholipids via COX, and producing prostaglandins specifically PGE_2_ and PGE_2_α, and level of lipoxygenase products may also increase in peritoneal fluid [33-35]. These prostaglandins and lipoxygenase product cause swelling and agony by the cumulative capillary permeability and liberating endogenous substances that stimulate pain nerve endings. NSAIDs cause inhibition of COX enzyme in the peripheral tissues and affect the transduction mechanism of key afferent nociceptors [36]. Our results of acetic acid-induced abdominal constriction assay demonstrated a prominent reduction in writhing reflux. The analgesic effect observed at 150 mg/kg dose was comparable with the NSAID standard drug diclofenac sodium (Table 4) [37,38]. These findings strongly recommend that AHM and AHE extracts of *A. hydaspica* have peripheral analgesic activity and their mechanisms of action may be mediated through inhibition of local peritoneal receptors via cyclooxygenase inhibition.

Thermal nociception models such as hot plat tests were used to evaluate the central analgesic activity. Both AHM and AHE showed significant (*p* < 0.001) analgesic effect in the hot plate test, implicating that plant extract may act as a narcotic analgesic (Table 5). Diclofenac sodium induces analgesic effect through activation of opioid receptors [39], and the apparent similarity between the results of extracts with standard diclofenac sodium, indicates that they might work in a same manner to reduce pain sensation as diclofenac sodium. The profound analgesic activity of *A. hydaspica* extracts might be due to the interference of their active principle (s) with the release of pain mediators, as the flavonoids increase the amount of endogenous serotonin or may interact with 5-HT2A and 5- HT3 receptors which may be involved in the mechanism of central analgesic activity [38]. Diclofenac sodium, fluoxetine (10 mg/kg) AHM, and AHE (150 mg/kg) raised the pain threshold level within 30 min of administration. AHM and AHE showed more pronounced analgesic effect at 150 mg/kg dose than fluoxetine (10 mg/kg); stronger effects of plant extracts than fluoxetine, is also in line with a proven concept that medicinal plants possess a combination of constituents, involving different mode (s) of action, offering synergistic effects with no side effects. However the difference in concentrations to achieve the maximum analgesic point could be explained by differences in the metabolic rate of each drug [40].

The presence of gallic acid, methyl gallate, 7-*O*-galloyl catechin, catechin, rutin and caffeic acid in the other species of the genus *Acacia,* their effect against COX and 5-lopoxygenase and the antipyretic action of the methanol extract of *A. modesta* leaves [10], supplemented the antipyretic, anti-inflammatory and analgesic activities of our tested extracts and validated its ethno-medicinal use as anti-inflammatory agent.

## Conclusion

To conclude, the methanol extract of *A. hydaspica* aerial parts (AHM) and its derived ethyl acetate fraction (AHE) were evidenced as a natural safe remedy for the treatment of pyrexia, pain and inflammation. Our current findings demonstrated mechanistic evidence for why indigenous people of Pakistan and India found it useful for inflammatory disorders. Interestingly the findings that extracts exhibited both peripheral as well as central analgesic effect provide a rationale for developing opioids alternative which leads to vomiting and nausea. The observed pharmacological activities might have been attributed to the presence of active principles. But there is still need for entailment of identification and acquaintance of molecular targets.
